# SeEn: Sequential enriched datasets for sequence-aware recommendations

**DOI:** 10.1038/s41597-022-01598-7

**Published:** 2022-08-04

**Authors:** Marcia Barros, André Moitinho, Francisco M. Couto

**Affiliations:** 1 LASIGE, Departamento de Informática, Faculdade de Ciências, Universidade de Lisboa, Lisboa, Portugal; 2 CENTRA, Departamento de Física, Faculdade de Ciências, Universidade de Lisboa, Lisboa, Portugal

**Keywords:** Data processing, Cheminformatics, Stars, Information technology, Scientific data

## Abstract

The recommendation of items based on the sequential past users’ preferences has evolved in the last few years, mostly due to deep learning approaches, such as BERT4Rec. However, in scientific fields, recommender systems for recommending the next best item are not widely used. The main goal of this work is to improve the results for the recommendation of the next best item in scientific domains using sequence aware datasets and algorithms. In the first part of this work, we present the adaptation of a previous method (LIBRETTI) for creating sequential recommendation datasets for scientific fields. The results were assessed in Astronomy and Chemistry. In the second part of this work, we propose a new approach to improve the datasets, not the algorithms, to obtain better recommendations. The new hybrid approach is called sequential enrichment (SeEn), which consists of adding to a sequence of items the n most similar items after each original item. The results show that the enriched sequences obtained better results than the original ones. The Chemistry dataset improved by approximately seven percentage points and the Astronomy dataset by 16 percentage points for Hit Ratio and Normalized Discounted Cumulative Gain.

## Introduction

Everyone appreciates a recommendation for a good movie or an interesting book. Why would it be different for researchers? An investigator studying the effect of chemical compounds in the creation of new drugs would be more than glad if a system recommended the next best match to their studies preferences. Several recent studies support this claim^1–5^. The goal of most of these studies is to obtain a recommendation for the response a drug will have in the patients, for example, realizing studies with patients’ cell cultures^1–4^. A different approach was followed by^5^, in which the recommendations are based on sentiment analyses of the patient’s reviews about drugs. Other scientific fields, such as Health^6^, and Astronomy^7–9^, are following the trend in seeking recommendations for finding new items of interest. The number of new scientific entities grows every day, requiring new tools for knowledge extraction. Recommender systems (RS) approaches to suit these situations since they can deal with large quantities of data and also provide personalized recommendations, according to the researchers’ preferences^10^. The main challenge is that there are few studies in recommender systems for scientific fields, primarily due to the lack of open-access datasets.

The recommendation of items has been a topic of interest in many fields, such as music, movies, e-commerce, and even scientific fields such as Chemistry and Astronomy. In some cases, the user/item interaction sequence is important since the next item of interest may depend on the previous ones. Despite a large number of studies on sequence-aware recommendation systems (RS)^11^, their use in scientific fields is not broad.

RS are, by definition, software tools and techniques that provide suggestions for items that are most likely of interest to a particular user, mostly used in the recommendation of movies, music, and e-commerce. There are two major approaches in RS, collaborative-filtering (CF) and content-based (CB) similarity^12^. CF uses only the users’ preferences as input for the recommendations, calculating the similarity between users. If John Smith and Jane Smith read the same article, they are similar users. Suppose Jane Smith reads a second article; it will be recommended to John Smith. The example refers to memory-based CF. Instead of directly calculating the similarity between the users, CF may be model-based, using machine-learning, for example, matrix factorization and deep learning, for predicting the ratings of unseen items. This approach has some challenges. It cannot deal with items without ratings or users who have not rated any item (cold start for new items and new users, respectively). In CB approaches, the recommendations do not depend on the similarity of the users but the similarity of the items. If Jane Smith reads an article, CB algorithms will recommend to her similar articles to the one she read without involving the preferences of other users. CB solves the problem of cold start for new items. However, for calculating the similarity between the items, we need a characterization of each item specified by a set of features. If the item is a movie, the features may be the genre, actors, and director. Then, we can use similarity metrics, such as cosine similarity, or machine-learning methods, for example, clustering approaches, to group the items by similarity. A particular type of similarity is the semantic similarity shared by the items. For calculating the semantic similarity of the items, we may use ontologies, which are vocabularies hierarchically organized^13,14^. Ontologies are widely used in Health and Life Sciences, with a large number of bio-ontologies being made available and maintained in the last few years, such as the Chemical Entities of Biological Interest (ChEBI)^15^, the Gene Ontology (GO)^16^, and the Disease Ontology (DO)^17^. Bio-ontologies are important since they help researchers to identify an entity unequivocally, and also enable the computation of the semantic similarity between the entities. Hybrid CF-CB approaches are used to get the best of both CF and CB. One of the methods used is the completion of the unknown ratings by calculating the similarity between the items that the user already rated and the unrated items (CB). The completed matrix is then used in CF approaches for finding the most similar users and providing the recommendations^18^.

All the recommendation approaches presented in the previous paragraph depend on information about the users’ preferences, usually as ratings. These ratings may be explicit, for example, through a stars classification system, or implicit, where the users’ preferences are collected from their activities, such as “user u watched movie b”. Open-access datasets with the users’ preferences are standard in movies, TV shows, music and e-commerce fields. For movies we have Movielens^19^ and Netflix^20^ datasets. In music, we find datasets provided by Spotify^21^, and for e-commerce, Amazon^22^ has been relentless in the promotion of these datasets, which translates in a large number of algorithms applied in these fields.

Standard and open-access datasets with information about users’ preferences are scarce in scientific fields, such as Chemistry and Astronomy. Thus, if we wish to develop an algorithm for recommending chemical compounds, we may lack access to a dataset with information about the past preferences of a group of users. Given this limitation, in Barros *et al*.^23^ we developed a new methodology called LIterature Based RecommEndaTion of scienTific Items (LIBRETTI) whose goal is the creation of <user, item, rating> datasets, related with scientific fields. These datasets are created based on the major resource of knowledge available in Science: scientific literature. The users are the authors of the publications, the items are the scientific entities (for example, chemical compounds or diseases), and the ratings are the number of publications where the author mentioned the entity.

**Fig. 1:**
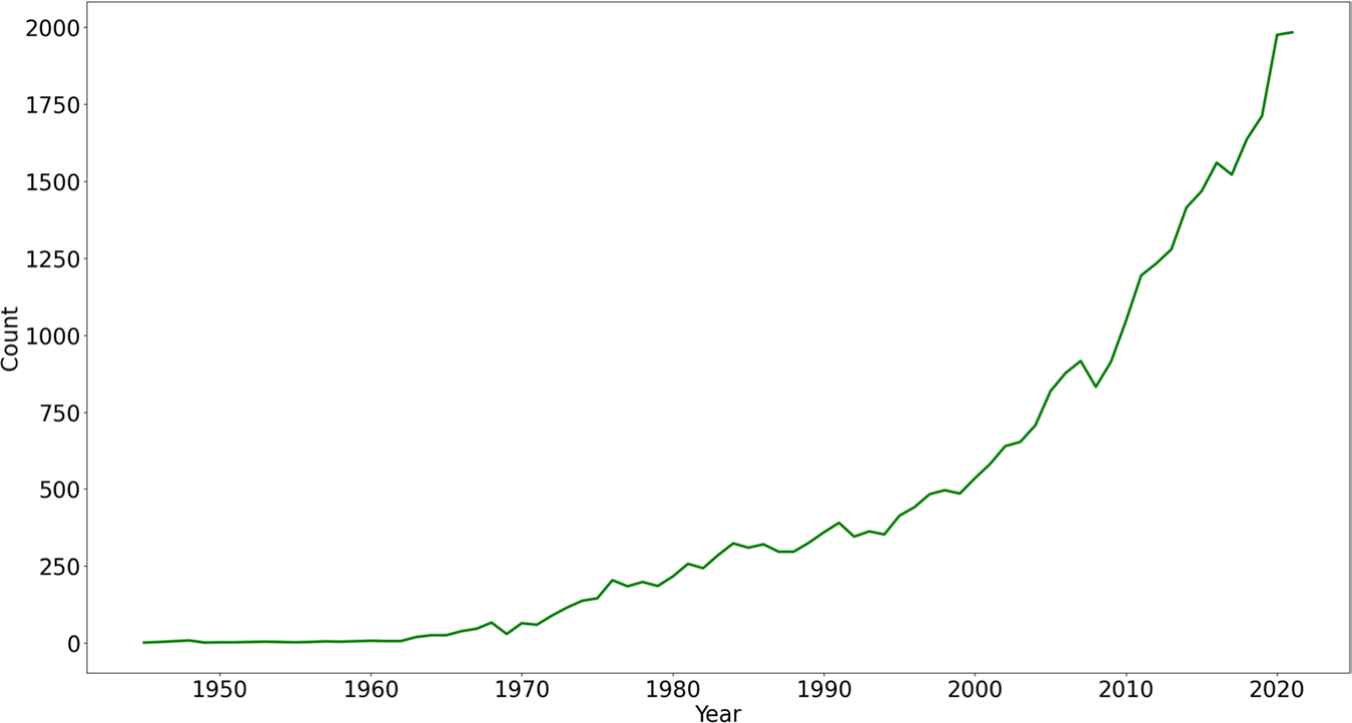
Paracetamol research articles by year in Pubmed.

Typical recommendation datasets have matrix format, with items in the columns, users in the rows, and the ratings being assigned to the pairs <user, item>. However, some situations require knowledge about the sequence in which the items were seen, especially in scientific fields, where scientific entities raise different degrees of interest to the researchers along the time. For example, according to Pubmed^24^, the chemical compound Paracetamol^25^ had a spike in the number of research articles in 2020, as shown in Fig. 1.

Sequence-aware recommendations arise to solve the problem where the order of the items is important to provide the recommendation of the next best item. Sequence-aware recommendations have been developed and applied to movies, music, and e-commerce, but to the best of our knowledge, not to scientific fields. There are already algorithms dealing with sequential recommendations. There are some standard baselines, e.g. selecting the most popular, or k-nearest-neighbours approaches. We also have non-deep learning approaches, such as matrix factorization and Markov chains^26^. Most recently, deep learning approaches have emerged as the state-of-the-art for sequence-aware recommendations, such as, GRU4Rec^27^, CASER^28^, SASRec^29^ and BERT4Rec^30^. The last one outperformed all the other algorithms. BERT4Rec is based on the famous BERT model^31^. BERT diverges from other deep learning algorithms in that it is bidirectional, reading the sequences from left to right and right to left. The first step of BERT4Rec is an embedding layer, where it combines the position and the item, and then several transformer layers. The transformer method is a deep learning model for Natural Language Processing (NLP), based on multi-head self-attention and another layer of position-wise feedforward. BERT4Rec has several transformer layers, and they are connected bidirectionally. For training, a subset of the items is masked in the sequence. The output is the probability of the candidate item being the next best item.

In many fields, such as movies and TV shows, it is possible to simulate implicit sequential datasets by using the timestamp associated with the <user, rating> pair and converting the ratings to binary (i.e. ratings of 0 or 1)^30^. In science, the available datasets do not have this information. Even our datasets created using the LIBRETTI methodology do not consider a timeline for the user’s interaction with the items. In this work, we recreated the LIBRETTI methodology to develop new datasets aware of the interaction sequence between user and item. Thus enabling the use of sequence aware algorithms for recommending the next best item to a researcher. The methods will be assessed in Chemistry and Astronomy for recommending chemical compounds and open clusters of stars, respectively.

Besides creating new sequential recommendation datasets, in this work, we also present a new methodology for sequence-aware recommendations in the fields of Chemistry and Astronomy, focused on the enrichment of the dataset, not on the improvement of the algorithm. The proposed methodology, called Sequence Enrichment (SeEn), employs a hybrid approach by adding to a sequence of items the n most similar items after each original item. The new sequence is then passed as input to state-of-the-art sequence-aware recommendation algorithms to improve the results compared with the not enriched or original sequence.

As seen previously, the sequence of the user-item interaction is essential in scientific domains. Thus, this study aims to prove that sequence-aware datasets are better for recommending the next best item in scientific fields.

The main contributions of this work are:

- A sequential dataset in the field of Chemistry for the recommendation of chemical compounds;
- A sequential dataset in the field of Astronomy for the recommendation of open clusters of stars;
- A new hybrid data-driven approach (SeEn) for sequence-aware recommendations.

## Methods

### Datasets

**Fig. 2:**
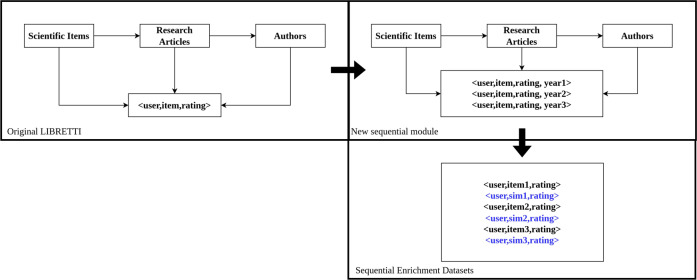
Scheme of the original LIBRETTI methodology vs new sequential module, and the enrichment of the sequential datasets.

For this work, we created two datasets from different scientific fields, one from Chemistry, where the items are chemical compounds, and another from Astronomy, where the items are Open Clusters of Stars. Both datasets were created according to the LIBRETTI methodology^23^ modified to develop sequences of items by user, ordered by the year of publication of the paper mentioning each item. The papers with the same year of publication are not ranked in a specific order^32^. Figure 2 shows the original scheme of LIBRETTI presented in Barros *et al*.^23^ vs the new sequential module. In both modules, LIBRETTI requires a list of scientific items and articles where the items are mentioned. Then, we extract the authors from the articles and create datasets of user (author), item (scientific entity), and rating (number of articles where the author mentioned the entity) for the original LIBRETTI. In the sequential module, the <user, item> interactions are ordered by the publication year of the article. The rating is always 1. Despite not bringing additional information to the recommendation algorithms, we have kept the rating columns since many recommendations tools require a rating column^33,34^. Figure 2 also shows a representation of the sequential dataset enriched with the most similar items, which will be better explained in Methods Section.

The Chemistry dataset, called chemicals Recommendation Matrix (**chERM**), is a dataset whose items are chemical compounds represented in the chEBI ontology. The first chERM dataset was created in^23^, and it was used in works^35,36^ for testing new algorithms for recommending chemical compounds. The original chERM dataset has the format of <user, item, rating>, the users being authors of research articles, the items being chemical compounds, and the ratings the number of articles in which a user mentioned the item. In the new chERM dataset (chERMSeq), the items are organized by year for each user, as represented in Fig. 7: Original chERMSeq. In these studies, the dataset chERMSeq was used to evaluate a new hybrid recommender algorithm based on the semantic similarity of the chemical compounds, calculated through the ChEBI ontology.

The Astronomy dataset, called astronomical Recommendation Matrix (**aRM**), is a dataset of Open Clusters of Stars, whose items were collected from the Dias catalogue of open clusters^37^. The method for creating this dataset is the same used in^23^, except for the updated list of Open Clusters. The new aRM dataset (aRMSeq) was created with the same method as chERMSeq.

Unlike datasets such as Movielens, chERMSeq and aRMSeq did not need to be converted to binary ratings since they are already implicit feedback datasets whose rating values are 1 (author mentioned entity in the article), or 0 (the author did not mention entity in the article). These implicit datasets will have drawbacks associated. For example:

- There is normally no negative feedback; we cannot know if the user did not like the item;
- There is associated noise; for example, authors participating in a research article with several items but only worked with one or two;
- The numerical value of the rating might only refer to a user’s preferences with some degree of confidence. For example, we assume that if a user watched a movie until the end, she/he liked it. If she/he left in the first moments, the item was not interesting to this user. We use this same principle: if a research author wrote about a paper, she/he had an interest in that item.

Despite the presented disadvantages, in the absence of datasets of explicit feedback, these are our best options for providing accurate recommendations. Next, we will present the new Sequential Enrichment approach developed in this work.

### Sequential enrichment approach

The recommendation of the next best item for a user is still a challenge. Sequential datasets are usually of implicit feedback, highly sparse, and with no negative feedback. In this work, we propose a solution for the datasets’ sparsity by introducing a hybrid sequential enrichment approach based on the similarity of the items.

**Fig. 3:**
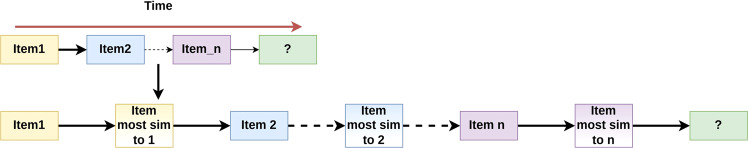
SeEn: Sequential enrichment approach general scheme.

Figure 3 shows the general pipeline of the SeEn approach. It consists in introducing after each item in a sequence its n most similar items, reducing the sparsity of the dataset. The new enriched sequence is then passed into sequence-aware recommender algorithms. We hypothesise that using the SeEn approach will improve the results of state-of-the-art algorithms.

The input of SeEn requires a recommendation dataset, where each user has a sequence of items with which the user already interacted, ordered by interaction time, for example, by year or timestamp. After each original item, the method introduces the n most similar items to the original into the sequence. For calculating the similarity, we need a knowledge source with the features of the items, which will depend on the field of study.

We may directly apply similarity metrics, such as cosine or Jaccard, to find the most similar items if we have numerical features. These metrics calculate the similarity between two vectors^38^. We may use semantic similarity to find the most similar items in other cases. The semantic similarity may be measured based on the semantic structure of an ontology, allowing to have the closeness in meaning between the entities^39^. Some known metrics are Resnik^40^, Lin^41^, and Jiang and Conrath (JC)^42^.

### Evaluation

This work is divided into two evaluation phases. First, we want to identify the best algorithm and prove that using sequential datasets to recommend the next best item results in better recommendations than when not considering the interaction sequence. Second, we want to evaluate if enriching the datasets with the n most similar items further improves the results.

For the first phase of the evaluation, we used the following algorithms for testing both chERMSeq and aRMSeq datasets:

- **The most popular (Most-Pop)** - The most popular recommendation algorithm is a basic algorithm that considers the items with the larger number of ratings and recommends the top@k to the user. The sequence of the items is not relevant.
- **Alternating Least Squares (ALS)** - ALS is a latent factor algorithm, specific for implicit feedback datasets, that addresses the confidence of a user-item pair rating, which goal is to minimize the least-squares error of the observed ratings by factorizing the rating matrix in user and item matrix. The order of the items is not relevant.
- **BERT4Rec** - BERT4Rec is a sequence-aware recommendation algorithm with state-of-the-art results in this field. The sequence of the items is relevant.

Table 1 shows the algorithms tested with which datasets. The Most-Pop and ALS algorithms were tested with the chERMSeq and aRMSeq datasets, but the order is not relevant in these cases. BERT4Rec was tested with the chERMSeq and aRMSeq not sequential, i.e., each user’s sequences were shuffled. BERT4Rec was also evaluated with the sequential chERMSeq and aRMSeq datasets.

**Table 1:** Evaluation of sequential datasets: algorithm and version of the dataset.

Algorithm	Dataset
Most-Pop	chERMSeq
	aRMSeq
ALS	chERMSeq
	aRMSeq
BERT4Rec	chERMSeq not seq
	aRMSeq not seq
	chERMSeq
	aRMSeq

To guarantee the quality of the datasets, we limited the minimum number of user/item interactions to 20. In aRMSeq, we also determined the maximum number of the sequence to 800 since one of the Transformers layer limitations of BERT4Rec is the maximum size of the sequence^43^.

For the second phase of the evaluation, we tested the SeEn approach. Table 2 shows the proceedings experiments. The selected algorithm was the BERT4Rec given its higher performance. The datasets used were the chERMSeq and the aRMSeq. Both were tested in their original sequential form and added the one, two, three, four, five, and ten most similar items to the sequence, as shown in the Sequential Enrichment Approach Section. We also tested adding random items to the sequence in the same proportion to evaluate the difference between adding random items or items selected according to the similarity. The original and the enriched sequences datasets were then used for training models with BERT4Rec^30^.

**Table 2:** Evaluation of the SeEn approach.

Dataset	Algorithms	SeEn
chERMSeq aRMSeq	BERT4Rec	Original
		Sim + 1
		Sim + 5
		Sim + 10
		Rand + 1
		Rand + 5
		Rand + 10

For both evaluation phases, the evaluation method was the leave-one-out, by hiding the last item in the sequence for test and the second-last for validation. We guaranteed that the last item was always the same, whether we were using the shuffled dataset or not. This is a typical method used to evaluate sequence-aware recommender systems since the goal is to predict the next best item. The evaluation metrics were the hit ratio (HR) (Eq. 1) and the Normalized Discounted Cumulative Gain (nDCG) (Eq. 2) at one, five, and ten. The hit ratio gives us the number of relevant items in a list of recommendations. The hit ratio will always be one or zero for each user because we only have one relevant item per user; thus, the item is or it is not in the top@k recommendations. The nDCG is an evaluation method that compares the ideal ranking of a test set (iDCG), with the ranking assigned by the recommendation algorithm (DCG - Eq. 3), allowing an evaluation about the position of the item in the top@k recommendation list.

1 $$HR=1-missRatio$$

2 $$nDCG=\frac{DCG}{iDCG}$$

3 $$DCG={\sum }_{i=1}^{n}\frac{relevanc{e}_{i}}{{{\rm{\log }}}_{2}\left.\left(i+1\right)\right)}$$

The framework used for the evaluation was the original Tensorflow implementation of Sun *et al*.^30^, available at https://github.com/FeiSun/BERT4Rec. For the original sequence, the max sequence used in chERMSeq was 50 and in aRMSeq was 100. For the enriched sequences, the max sequence was 50 + (50 × *n*) for chERMSeq and 100 + (100 × *n*) for aRMSeq, where n is the number of similar items added to each original item in the sequence. The models were trained on a NVIDIA Tesla P4 GPU with a batch size of 256.

### SeEn Item-item similarity methods

As already mentioned, different fields depend on different features for calculating the similarity between the items. In the Chemistry case study, we are dealing with chemical compounds. There are several methods for measuring the similarity between chemical compounds, such as structural similarity and semantic similarity. Some studies suggest that the semantic metrics are better for finding the similarity between the compounds^44–46^. In^36^, the authors used the semantic similarity between the items to create a hybrid semantic recommender system for chemical compounds. They tested the metrics Resnik^40^, Lin^41^, and Jiang and Conrath (JC)^42^. The authors also provided an open-access database with more than 128k compound-compound similarity for all the three metrics, which was created using the framework DiShIn^39,47^. The Lin metric results were one of those with better results, which is why we are using it in this work.

In the Astronomy case study, for calculating the similarity between the open clusters of stars, we used the features of the Gaia ESA’s dataset^48^. Gaia is an astronomical mission with the goal of collecting information about the stars in the Milky Way. The dataset is in the third release, and it has more than 1.9 million stars. We used the stars in Gaia mapped to each open cluster for this work^49^. Then we calculated the mean of the features for each open cluster, and the mean of the features was used for calculating the similarity, using the Cosine similarity (Eq. 4, where x and y are two non-zero vectors). For the tests presented in this work, we used the features related to the location: longitude, latitude and parallax. The output was a dataset of cluster-cluster similarity with approximately 1.5 million entries.

4 $$cosine\;similarity({\boldsymbol{x}},{\boldsymbol{y}})=\frac{{\boldsymbol{x}}\cdot {\boldsymbol{y}}}{| | {\boldsymbol{x}}| | \cdot | | {\boldsymbol{y}}| | }$$

## Results

This section presents the results for the new sequential datasets created through the LIBRETTI methodology and the results for the new sequential enrichment (SeEn) approach. The original LIBRETTI allows the creation of standard <user,item,rating> recommendation datasets from scientific domains. The users are authors from research articles, the items are scientific entities mentioned in the articles, and the ratings are the number of articles where a user mentioned an entity. No timeline is regarded. The new sequence-aware recommendation datasets follow the same user and item approach; however, for each user, the items are ordered by the year of publication of the article mentioning the item. The Section Datasets shows how sequence-aware vs non sequence-aware algorithms behave and also how sequence-aware algorithms behave when provided with sequential vs non-sequential datasets as input.

The Section SeEn presents the results related to the new recommendation approach, which enhances the datasets with the most similar items to the ones the user already interacted with and uses the new enriched dataset as input to BERT4Rec, a sequence-aware recommendation algorithm. Both parts of this work were tested in the scientific fields of Astronomy and Chemistry.

### Datasets

In this section, we present the results for the sequence aware recommendation datasets in the fields of Chemistry (for recommending chemical compounds) and in the field of Astronomy (for recommending open clusters of stars). Short examples of both datasets are presented in Table 3. The Chemicals Recommendation Matrix sequence (chERMSeq) has as columns user, item, rating and year. The user is an ID assigned by us, corresponding to an author’s name. The item is the ID of the chemical compound in the ChEBI ontology. For example, the ID 18357 corresponds to (R)-noradrenaline^50^. The rating is always one, and the year corresponds to the publication year of the article mentioning the chemical compound. The astronomical Recommendation Matrix sequence (aRMSeq) dataset also has the columns user, item, rating and year, and an extra with the item name. This happens because, in this case, the column item corresponds to an ID assigned by us. Thus it may be helpful also have the item name.

**Table 3:** chERMSeq and aRMSeq examples.

chERMSeq				
User	Item	Rating	Year	
378	18357	1	1984	
378	71045	1	2010	
378	131855	1	2015	
378	142842	1	2016	
**aRMSeq**				
**User**	**Item**	**Rating**	**Item name**	**year**
25	696	1	NGC_2264	2005
25	625	1	Melotte_22	2011
25	769	1	NGC_2682	2013
25	894	1	NGC_6811	2020

Table 4 shows the statistics of the new datasets. The chERMSeq has fewer ratings and more items than aRMSeq; thus, it is sparser. The aRMSeq dataset has longer sequences than chERMSeq. The minimum size of the sequences for both datasets is 20 to avoid users with few ratings, also known as cold start. Table 5 shows the total number of users for the chERM and aRM original datasets, before any filtering. In the chERM original dataset, 67% of the users have only one item rated. These users are not suitable for testing recommendation algorithms. This percentage is lower in the original aRM dataset, with 29% of the users having only one rating. For a matter of standardization, we filtered all the users with less than 20 items rated in both datasets. This leads to a major decrease in the number of users, but we guarantee datasets with high quality for testing and evaluating recommendation algorithms.

**Table 4:** chERMSeq and aRMSeq datasets statistics.

Dataset	Total	Users	Items	Min seq	Max seq	Mean Seq	Year range	Type of items	Sparsity
chERMSeq	131k	2.5k	16k	20	783	53.43	1951–2019	Chemical compounds	99.68
aRMSeq	276k	2.7k	1k	20	4314	101.25	1998–2020	Open clusters of stars	90.15

**Table 5:** chERMSeq and aRMSeq original datasets total number of users, number of users with only one item rated, number of users with 20 or less items rated.

Dataset	Total of users	Users with only one item rated	Users with 20 or less items rated
chERMSeq original no filters	193k	129k (67%)	190k (98%)
aRMSeq original no filters	16k	4.5k (29%)	13k (84%)

**Fig. 4:**
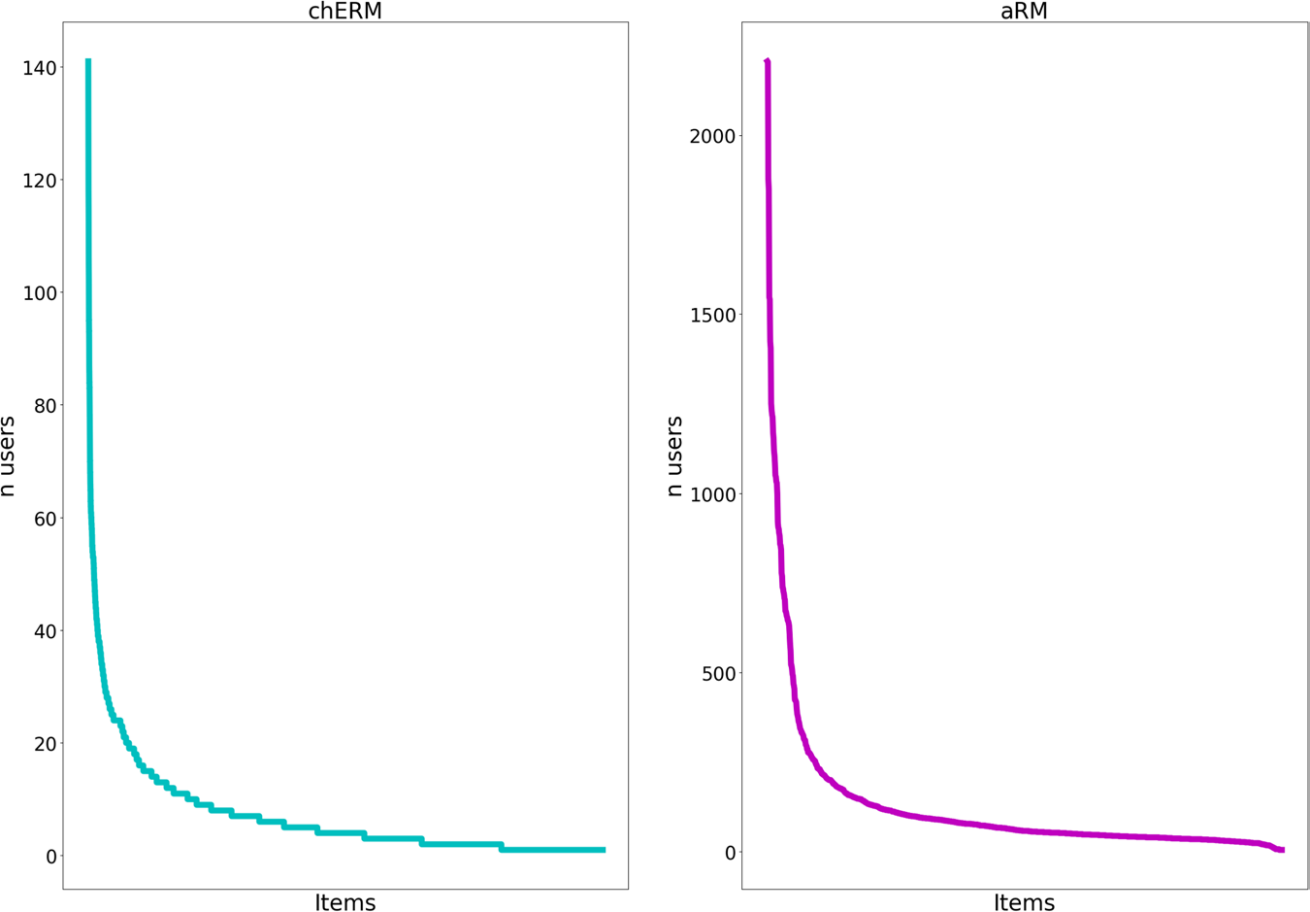
chERMSeq and aRMSeq number of users rating each item.

**Fig. 5:**
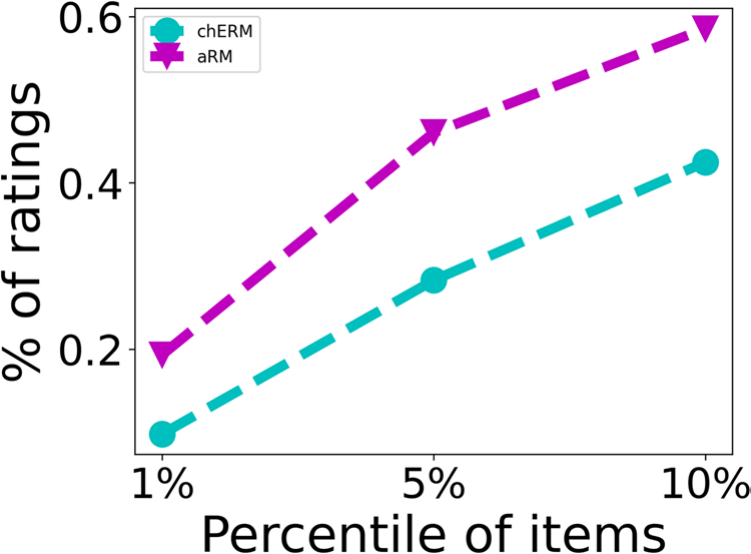
Distribution of ratings by percentile of item at 1, 5 and 10%.

Figure 4 presents the distribution of the ratings by each one of the items in chERMSeq and aRMSeq datasets, i.e., the number of users (n users) who rated that specific item. The plots show the typical long-tail phenomenon where a small number of items has the majority of the ratings, whereas a large number of items have only a few ratings. For chERMSeq, the item with more users (141) is the CHEBI:17754 (glycerol). For aRMSeq, the item with more ratings (2206) is the Melotte_22, also known as the Pleiades. Analysing both plots, despite the similar number of users in the datasets, the aRMSeq dataset concentrates a much larger number of ratings in a small number of items than the chERMSeq dataset. This can be better observed in the plot of Fig. 5, where we present the results for the distribution of the ratings by 1, 5 and 10% of the most rated items. The results show that in the chERMSeq dataset 1% of the items receive 9% of the ratings. In aRMSeq dataset, 1% of the items get 20% of the ratings.

Next, we present the results related to the analysis of different recommendation algorithms applied to chERMSeq and aRMSeq datasets (See Table 1). The goal is to evaluate how sequence-aware recommender algorithms, such as BERT4Rec, improve the recommendations of the next best item and how sequential or not sequential data affect these results.

**Fig. 6:**
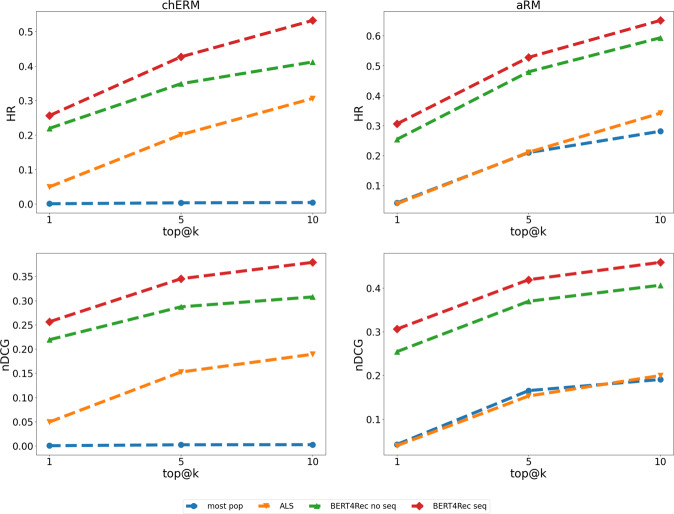
Analysis of the results for the recommendation of chemical compounds (chERMSeq) and open clusters of stars (aRMSeq), with the algorithms most pop, ALS, BERT4Rec no seq, and BERT4Rec seq, for the metrics of Hit Ratio (HR) and Normalized Discounted Cumulative Gain (nDCG) @k.

Figure 6 shows the plots for the recommendation algorithms most popular, Alternating Least Squares (ALS), BERT4Rec using non-sequential datasets, and BERT4Rec using sequential datasets. The most popular and the ALS algorithms are CF algorithms and do not consider the sequence of the items. The first always recommends the k items with the most ratings, and the former is a latent factor algorithm based on the similarity of the users. BERT4Rec is a state-of-the-art sequence-aware algorithm based on neural networks. The algorithms were evaluated using the chERMSeq and the aRMSeq datasets. The evaluation metrics were the Hit Ratio (HR) and the Normalized Discounted Cumulative Gain (nDCG) @ 1, 5 and 10.

The analysis of Fig. 6 shows that for the chERMSeq dataset, the algorithm most popular achieved the worst results, as expected, followed by an improvement of more than 20 percentage points for ALS. BERT4Rec surpasses this result when tested with the not ordered sequences. BERT4Rec achieves the best results with the sequence dataset. For the aRMSeq dataset, the most-pop and ALS algorithms achieved similar results, followed by BERT4Rec with the non-sequential dataset and BERT4Rec with the sequential dataset.

### SeEn

The datasets presented in the previous section have sparsity levels superior to 90%, which may lead to inferior recommendation results. To improve the quality of the datasets, we developed the SeEn approach presented in this work. Table 6 shows the sparsity levels of the different datasets created through SeEn methodology. We can see that the sparsity decreases with the increase of the number of added items to the sequence, especially in the aRMSeq cos + 10 dataset.

**Table 6:** chERMSeq and aRMSeq enriched datasets statistics.

Dataset	Total	sparsity
chERMSeq sim_lin + 1	264k	0.993
chERMSeq sim_lin + 5	784k	0.981
chERMSeq sim_lin + 10	1.434 M	0.966
aRMSeq sim_cos + 1	547k	0.834
aRMSeq sim_cos + 5	1.633 M	0.535
aRMSeq sim_cos + 10	2.990 M	0.151

**Fig. 7:**
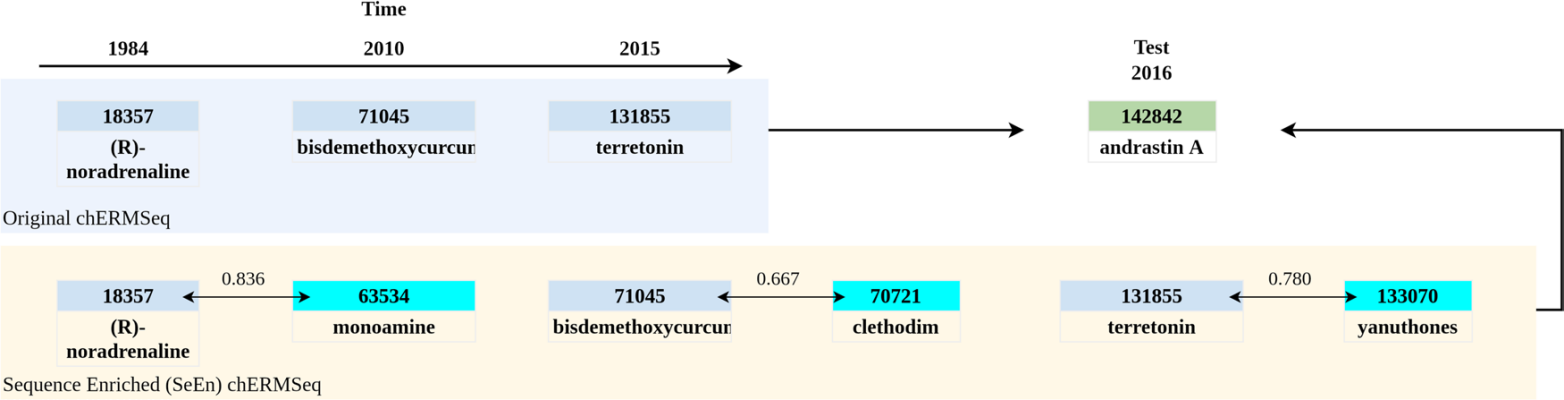
Sequential enrichment example. (R)-noradrenaline is 0.836 similar to monoamine, bisdemethoxycurcumin is 0.667 similar to clethodim, and terretonin is 0.780 similar to yanuthones.

Figure 7 shows a real example of sequential enrichment for a sequence of chemical compounds. In the case presented, the user has three items in the train set, (R)-noradrenaline, bisdemethoxycurcumin, and terretonin, ordered by year. SeEn enriched chERMSeq has added to the sequence the most similar chemical compounds. The goal is to recommend the compound andrastin A (test item).

Table 7 shows the results obtained using BERT4Rec, for the original dataset chERMSeq and aRMSeq, and these datasets with sequences enriched with the SeEn approach. In addition to the results for sim + 1, sim + 5 and sim + 10, we also included the results for sim + 2, sim + 3, and sim + 4, for a better perception of the evolution of the results. For the chERMSeq dataset, the Sim_lin + 1 obtained the best results for both HR and nDCG, increasing the original results by approximately seven percentage points. The values of the evaluation metrics decrease with the increase of n. For the aRMSeq dataset, the best results were achieved when enriching the sequence with one most similar items, increasing the results by 16 percentage points. In general, the random achieved worse results than the original, proving that introducing similar items is better than introducing random items in the sequence.

**Table 7:** chERMSeq and aRMSeq SeEn results for HR and nDCG @ 1, 5, and 10.

Dataset	Hit@1	nDCG@1	Hit@5	nDCG@5	Hit@10	nDCG@10
cheRMSeq original	0.2562	0.2562	0.4268	0.3451	0.5326	0.3789
Sim_lin + 1	**0.3293**	**0.3293**	**0.4741**	**0.4058**	**0.5560**	**0.4323**
Sim_lin + 2	0.3126	0.3126	0.4638	0.3920	0.5453	0.4184
Sim_lin + 3	0.3186	0.3186	0.4545	0.3992	0.5388	0.4264
Sim_lin + 4	0.2959	0.2959	0.4419	0.3701	0.5242	0.3966
Sim_lin + 5	0.2828	0.2828	0.4339	0.3611	0.5482	0.3885
Sim_lin + 10	0.1980	0.1980	0.3090	0.2537	0.3929	0.2806
rand + 1	0.2087	0.2087	0.4097	0.3273	0.5060	0.3584
rand + 5	0.2207	0.2207	0.3532	0.2885	0.4431	0.3174
rand + 10	0.1256	0.1256	0.2036	0.1667	0.2645	0.1863
ARMSeq original	0.3059	0.3059	0.5279	0.4188	0.6513	0.4585
Sim_Cos + 1	**0.4680**	**0.4680**	**0.6801**	**0.5809**	**0.7718**	**0.6107**
Sim_Cos + 2	0.3926	0.3926	0.6450	0.5249	0.7561	0.5611
Sim_Cos + 3	0.3030	0.3030	0.5760	0.4439	0.7211	0.4912
Sim_Cos + 4	0.2697	0.2697	0.5596	0.4206	0.7029	0.4668
Sim_Cos + 5	0.2896	0.2896	0.5417	0.4189	0.6942	0.4680
Sim_Cos + 10	0.2469	0.2469	0.4869	0.3686	0.6330	0.4159
rand + 1	0.1866	0.1866	0.2652	0.2074	0.3348	0.2298
rand + 5	0.1552	0.1552	0.2523	0.2091	0.2726	0.2343
rand + 10	0.1955	0.1955	0.3097	0.2290	0.3579	0.2440

**Fig. 8:**
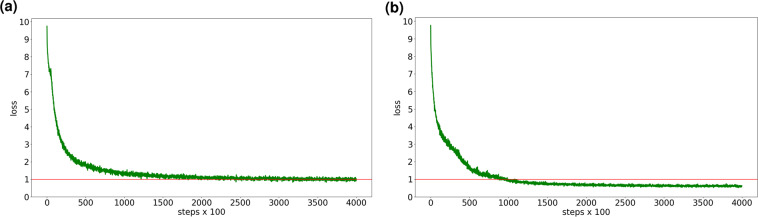
Loss for chERMSeq original dataset (**a**) and chERMSeq Sim_lin + 1 dataset (**b**). Horizontal red line: loss = 1.

Figure 8 shows the loss values for the original chERMSeq dataset and the chERMSeq dataset enriched with Sim_lin + 1 for the models trained with the BERT4Rec algorithm. The horizontal red line represents the loss equal to 1. Analysing the plots for the loss value, we see that the model trained with the chERMSeq Sim_lin + 1 dataset achieved lower loss values (bellow 1) within fewer steps (150000 vs 70000 for the original and SeEn, respectively). With this, we conclude that with SeEn we create better models with fewer training steps.

## Discussion

To overcome the challenge of the lack of sequence-aware open-access recommendation datasets in scientific domains, we developed a new module for an existent method (LIBRETTI) that creates recommendation datasets suitable for providing recommendations in scientific fields. We assessed the adaptation of the method in the fields of Chemistry and Astronomy. The results were two datasets, chERMSeq and aRMSeq, for recommending chemical compounds and open clusters of stars, respectively. The larger number of items in the chERMSeq dataset (16k vs 1k) and the similar number of users (2k) results in a sparser rating matrix for chERMSeq (Table 4).

The distribution of the ratings by the items follows the typical long tail of recommendation datasets, with few items having the majority of the ratings (Fig. 4), which may influence the recommendation algorithms. The plots in Fig. 6 show how the recommendation algorithms provide evidence that sequence aware algorithms are better for the recommendation of the next best item in chERMSeq and aRMSeq. When evaluating the most popular algorithm in chERMSeq, it got values close to zero, suggesting that the users do not share a large percentage of the most rated items. The behaviour with aRMSeq is different, with the most-pop algorithm achieving results similar to ALS. Looking at Fig. 5, we can see that the 10% of most popular items have 60% of the ratings in the aRMSeq dataset, against 40% in chERMSeq.

The major goal of this work was to prove that sequence aware recommendation datasets are needed for better next item recommendations. The span of results achieved by the assessed algorithms shows that algorithms not tailored for sequence-aware recommendations (most-pop and ALS) perform worse than algorithms designed for sequence recommendations (BERT4Rec) by a margin of more than 20 percentage points. BERT4Rec obtains better results when provided with the datasets with the items ordered by year (chERMSeq and aRMSeq seq) than randomly shuffled. chERMSeq improves the outcomes of BERT4Rec by 12 percentage points in the HR metric and seven percentage points in the nDCG @10. aRMSeq obtained approximately five more percentage points for HR and nDCG@10 than the shuffled version.

Following the evaluation of the datasets, we tested a new approach to address the problem of lack of knowledge in a single sequence. We called sequence enrichment (SeEn) to this approach. SeEn consists of adding to the sequence of items of each user the n most similar items after each item, as exemplified in Fig. 7. Depending on the field, the method for finding the most similar items will differ due to the specific characteristics and data available. For the case study in Astronomy, we used the cosine similarity, and for the case study in Chemistry, we used the semantic similarity with the metric Lin. Comparing the results presented in Table 7, for both HR and nDCG evaluation metrics, the SeEn datasets enriched with +1 most similar item obtained better results than the original dataset. The results for sim +2, +3 e +4 are generally higher than the original but smaller than with +1. After that number, the entropy introduced into the sequences leads the models to less accurate predictions. The improvement of the sparsity levels (Table 6) does not override the introduction of excessive noise in the datasets. For example, the aRMSeq sim_cos +10 has a sparsity lever of 0.151, meaning that the majority of the items are somehow related to all the users, but the results of the recommendation algorithms are much lower.

Measuring the advantages and disadvantages of the SeEn, we see that the approach seems to improve the results of BERT4Rec, recommending the right next item in the first position of the list of recommendations. We believe this is because it provides the recommendation of new items, for example, if we are trying to recommend an item from this year, if it does not exist in the original dataset, it will never be recommended when the model is trained with the original datasets. A possible disadvantage of this approach is the increase in the size of the sequence, which is a problem for algorithms such as BERT4Rec, with a computational complexity of O(n2d), quadratic with the length n.

RS and their applications are highly dependent on the field of study. Despite that, the SeEn approach proved to be suitable for two scientific field with several differences between each other, Chemistry and Astronomy. Observing the results presented in this study, we may conclude that there is a need for sequence recommendation datasets in scientific items. The enrichment of these datasets leads to better results in BERT4Rec, a state-of-the-art recommendation algorithm.

This study leaves several open possibilities. First, the SeEn approach may be an ally against the cold start challenge when a user has few rated items. The increment of the sequences with the most similar items may allow the algorithms to recommend items with a higher value for the users. Second, in this study, we consider all the items as having the same rating value (rating = 1). Further studies can be conducted to evaluate how different values of ratings influence the algorithms’ results. The different ratings may be, for example, 1 for the original rated item and the value of the similarity for the items added through SeEn. Looking at Fig. 7, the rating of this user for (R)-noradrenaline would be 1, and for monoamine would be 0.836. The new rating system would help with the challenge of negative ratings for the implicit feedback data.

## Data Availability

All the data used on this work is available at Barros, Marcia (2022): seen_datasets. figshare. Dataset. 10.6084/m9.figshare.18857549.v1^32^.

## Code availability

The code used for creating the datasets is available at: https://github.com/lasigeBioTM/SeEn.
